# Supplementary material for: Carotenoid Cleavage Dioxygenase Gene CCD4 Enhances Tanshinone Accumulation and Drought Resistance in Salvia miltiorrhiza
Source: Int J Mol Sci. 2024 Dec 9;25(23):13223. doi: 10.3390/ijms252313223 (PMC11642500; doi:10.3390/ijms252313223)
Supplement: Supplementary file 1 [file ijms-25-13223-s001.zip › ijms-3300146 - supplementary.pdf]

International Journal of
Molecular Sciences

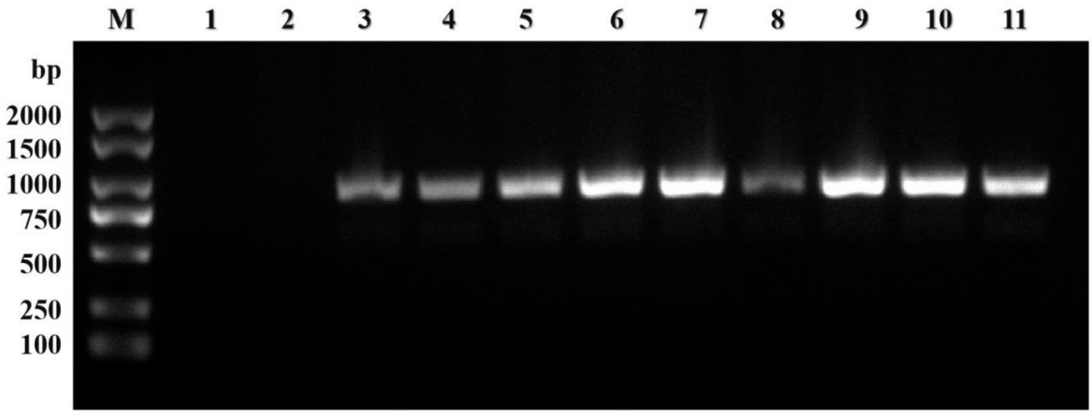

Figure S1. The *CaMV35S* promoter were confirmed by PCR in blank control (line 1), negative control (line 2), positive control (line 3), and eight transgenic overexpression strains (line 4-11).

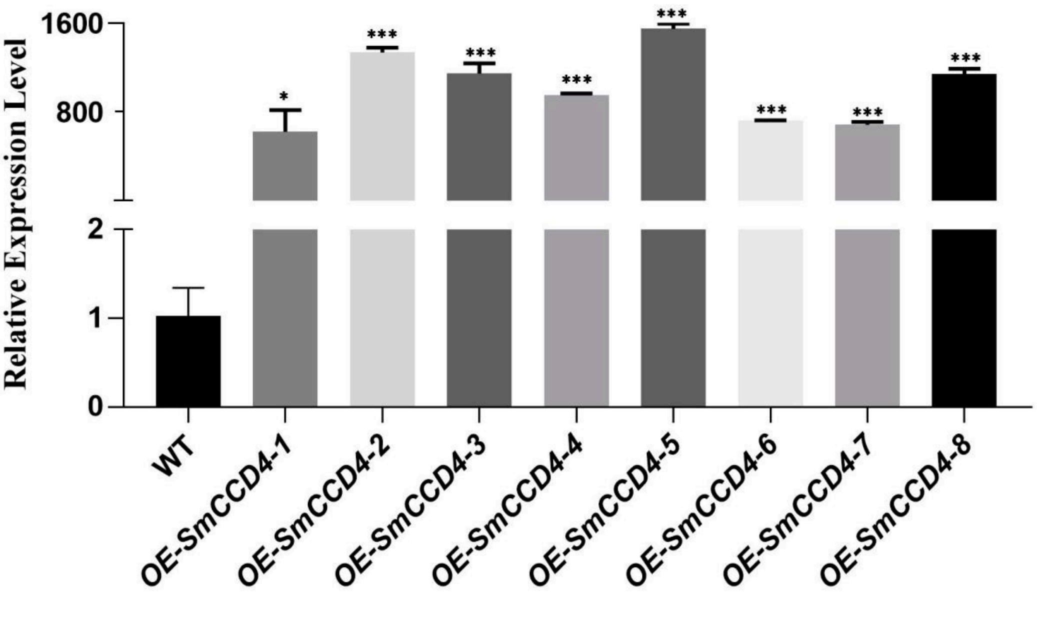

Figure S2. Identification of expression levels of the eight OE positive strains in *S. miltiorrhiza*. (*** $p < 0.001$; * $p < 0.05$).
